# Perceived communication efficacy and unmet needs for chemotherapy-associated symptom management in patients with lung and colorectal cancer: a cross-sectional study

**DOI:** 10.1186/s12904-024-01376-9

**Published:** 2024-03-13

**Authors:** Kai Zeng, Yaping Zhong, Xiaofang Chen, Lili Zhang

**Affiliations:** 1 School of Nursing, Southern Medical University, No.1023-1063 Shatai South Road, Baiyun District, Guangzhou, 510515 China; 2 Peter MacCallum Cancer Centre, Melbourne, Australia; 3 The Third Affiliated Hospital, Southern Medical University, Guangzhou, China

**Keywords:** Unmet needs, Symptom management, Communication efficacy, Patients with cancer

## Abstract

**Background:**

Understanding cancer patients’ unmet needs for chemotherapy-related symptom management will assist clinicians in developing tailored intervention programs. Little is known about the association between perceived communication efficacy and unmet care needs for symptom management in patients with lung and colorectal cancer.

**Objectives:**

To examine the unmet care needs for symptom management of patients with lung and colorectal cancer and their association with perceived communication efficacy.

**Methods:**

A cross-sectional survey was conducted in a tertiary hospital in China from July to November 2020. A convenience sample of 203 patients with lung and colorectal cancer undergoing chemotherapy completed survey questionnaires, including the MD Anderson Symptom Inventory Scale and the Perceived Efficacy in Patient‒Physician Interactions Scale.

**Results:**

Approximately 43% of participants had at least one symptom with unmet needs. Fatigue was reported as the symptom with the highest occurrence (66%), the highest demand for supportive care (36%), and the highest prevalence of unmet needs (19%). Low levels of perceived communication efficacy independently predicted participants’ unmet needs for symptom management (*β*=-0.13, *p* = 0.011).

**Conclusions:**

This study highlights the necessity of introducing clinical assessment tools and guidelines to address fatigue and other chemotherapy-induced symptoms in patients with lung and colorectal cancer. Clinical programs designed to actively engage cancer patients to voice their needs and strengthen their communication efficacy are also warranted.

## Key messages

i) What was already known? Patients with lung and colorectal cancer are prone to suffering from extremely high symptom burden associated with chemotherapy. Patient communication efficacy plays a crucial role in successful symptom control.

ii) What are the new findings? Unmet needs for chemotherapy-associated symptom management are prevalent in patients with lung and colorectal cancer in China. Perceived communication efficacy by patients with lung and colorectal cancer independently predicted their unmet needs for symptom management.

iii) Implications for practice, theory or policy: A patient-centered communication approach that fosters respect and encourages information sharing should be adopted clinically. Clinical training programs that strengthen core communication skills in healthcare professionals in direct patient-facing roles are warranted.

## Background

According to the latest estimates by the World Health Organization, [1, 2] cancer was identified as the leading cause of death in people aged 70 years or less in 112 countries. In the United States, totally 609,820 deaths are estimated to occur in 2023 [3]. In China, the cancer death rate continued to increase in recent years [4]. With recent advances in cancer treatment, particularly the widespread use of chemotherapy, the overall survival rates and prognosis of cancer have significantly improved [3]. Nevertheless, chemotherapy-induced symptom clusters are also widely reported in patients with cancer [5–7]. Patients with lung and colorectal cancer are frequently suffering from extremely high symptom burden associated with chemotherapy that adversely influences their functional status, psychological wellbeing, and quality of life [8]. 

It is widely recognized that effective symptom management is an essential component of palliative care that can alleviate the physical and psychological suffering of cancer patients, thus to improve quality of life during their treatment [9, 10]. However, several symptoms are often undervalued and overlooked by clinicians, which may result in unmet care needs in this patient population. Unmet care needs for symptom management are frequently reported in patients with cancer [11]. The definition of unmet care needs for symptom management is broadly considered as perceived discrepancy between expectations and the actual receipt of supportive care to relieve cancer-related symptoms [12]. Understanding unfulfilled supportive care needs in patients with cancer will reflect the deficit of symptom management, [13, 14] and help clinicians develop tailored symptom management plans for patients by prioritizing symptom treatment. This is particularly important in patients with lung and colorectal cancer due to the high prevalence of cancer occurrence, [1, 2] relatively rapid disease progression, and insidious symptom trajectory [8]. 

Unmet care needs for symptom management during chemotherapy were found to be highly variable depending on the cancer type, stage, and treatment regime. Physical symptoms, such as fatigue, pain, dyspnea, decreased appetite, and other gastrointestinal symptoms, were common in patients with advanced cancer, as reported by Wang [15] et al. In addition, psychological symptoms, such as sadness, depression, fear, and anxiety, were also found to be prevalent in patients with various cancer diagnoses, particularly in those receiving dose-intense chemotherapy [15–17]. Previous studies examining the supportive care needs of lung [18] and colorectal cancer [19] patients highlighted that fatigue, nausea, and vomiting were symptoms with a high occurrence but were often undermanaged, significantly reducing patients’ quality of life. The preponderance of evidence regarding unmet care needs for chemotherapy-related symptom management was from studies of patients with mixed cancer diagnoses, [20] and only some studies specifically focused on lung and colorectal patients [21, 22]. Moreover, prior research examining lung and colorectal patients’ unmet supportive care needs has mainly targeted patients in survivorship or during post-treatment phases [14, 18, 19]. These data are more reflective of unmet supportive care needs in primary care settings and rather than acute-care settings.

Effective patient-clinician communication is recognized for its positive impact in enhancing patient satisfaction in healthcare [23]. Applying the Dynamic Symptoms Model, Brant [24] and his colleagues emphasized the central role that communication between patients and clinicians plays in the quality of symptom management. Given the important link between the quality of care and patients’ satisfaction with their needs, [16] it is reasonable to hypothesize that patient-clinician communication may influence the unmet needs for symptom management among patients with cancer. The body of literature has also demonstrated the crucial role of effective patient‒physician communication in successful symptom control in patients with breast cancer and ovarian cancer [25, 26]. Patient perception of communication efficacy in healthcare is defined as one’s perceived confidence in their ability to source information and report concerns when interacting with clinicians, including nurses, physicians, and other members of allied health [27]. Such ability of patients may be an indicator of the quality of patient-clinician communication and may potentially influence their healthcare outcomes [28]. In patients with cancer, barriers to effective communication about symptom experience and supportive care needs have been investigated in previous studies [26, 29]. A 2019 survey conducted in the United States found that nearly 60% of patients with cancer were unwilling to discuss their symptoms with their clinicians during their medical visits for various reasons, including fear of additional treatment, uncertainty about treatment efficacy, and fear of being considered “a complainer” [29]. A comparative survey of Chinese American patients with breast cancer and their non-Hispanic American counterparts revealed that Chinese patients had significantly lower communication efficacy when interacting with physicians [30]. Active disclosure of personal information was not encouraged in the Chinese collectivist culture, which emphasizes group consensus over individual autonomy [31]. When this occurs in cancer care, it is likely that patients’ unreported symptoms may lead to their care needs being unnoticed and unaddressed, which adversely affect their prognosis and quality of life.

Previous studies have demonstrated a significant negative relationship between communication self-efficacy and perceived symptom burden in patients with breast and prostate cancer [25, 32]. To the best of our knowledge, no study to date has investigated the association between perceived communication efficacy and unmet symptom management needs in patients with lung and colorectal cancer undergoing chemotherapy. Our study is designed to fill this knowledge gap. Data generated from this study have important implications for clinicians to guide patients with lung and colorectal cancer to voice their needs for symptom management more effectively in acute-care settings or in palliative care.

## Methods

### Study design and settings

The study adopted a cross-sectional design. Sampling took place in a tertiary hospital in Guangzhou, China, from July to November 2020. This hospital had a capacity of 3600 beds, with approximately 100 inpatient beds allocated for the admission of patients with lung and colorectal cancer.

### Participants and data collection

This study was approved by the institutional review boards (2020-003). Participants were recruited by convenience sampling. Adult patients (≥ 18 years of age) who were diagnosed with lung or colorectal cancer and were hospitalized to receive chemotherapy were included. Patients who were illiterate, emotionally labile due to disease or personal factors, as assessed by nurses, or experiencing communication difficulties were excluded. Eligible potential participants were approached by nurses. An explanatory note detailing the purpose and procedures of this study was provided to each participant. Participants were also debriefed about the voluntary nature of their participation and were assured of confidentiality. A written informed consent was obtained prior to data collection. The sample size was calculated using G-power [33]. A sample size of 127 was considered sufficient, with α set at 0.05, 1-β at 0.80, effect size at 0.15, and the number of predictors at 12.

### Survey instrument

A paper-based self-administered questionnaire was developed with reference to previous studies [31, 32] and distributed to participants. The questionnaire included two scales, the Chinese version of the MD Anderson Symptom Inventory (MDASI-C) and the Chinese version of the Perceived Efficacy in Patient‒Physician Interactions Scale (PEPPI-C). Both scales have been validated for use in patients with cancer [34, 35] and had good Cronbach’s alpha values in the current sample (0.91 for MDASI-C and 0.98 for PEPPI). In addition to these, patient demographic characteristics, including age, gender, marital status, level of education, and household income, were also assessed (5 items). Participant clinical information was obtained by referring to the hospital electronic medical records. Clinical information included cancer stage, comorbidity, tumor metastases, chemotherapy cycles, and length of hospital stay (5 items).

#### Chinese version of the MD Anderson Symptom Inventory (MDASI-C)

There were 13 symptoms included in the MDASI-C scale for assessment: pain, fatigue, nausea, sleep disturbance, distress, shortness of breath, memory problems, decreased appetite, drowsiness, dry mouth, sadness, vomiting, and numbness [34]. Symptom severity was measured using an 11-point Likert scale ranging from 0 (symptom “absent”) to 10 (symptom “as bad as you can imagine”). A higher score indicates greater symptom severity, with a total score ranging between 0 and 130. In this study, participants first rated the severity of each symptom. For the symptoms that occurred, participants were further asked to identify whether their care needs for the respective symptom were satisfied during hospitalization. Sample questions were as follows: “During hospitalization, did you expect to get any help from clinicians to relieve your pain?” If the answer given was yes, then a follow-up question was “Were your needs for pain management met or unmet during hospital stay?” For each participant, the number of symptoms that required supportive care (range: 0–13) and the number of symptoms for which their care needs were unfulfilled during hospitalization (range: 0–13) were determined.

#### Chinese version of perceived efficacy in patient–physician interactions scale (PEPPI-C)

Patient perception of communication efficacy was measured by the 10-item PEPPI-C scale [27]. Participants rated their perceived level of confidence in their ability to ask questions, source information, report concerns, obtain attention, and seek health care from clinicians. A sample item is “How confident are you in your ability to have a doctor or nurse to pay attention to what you have to say?” We changed the term “physician” in the original scale to “clinician” to match the present research purpose. The term “clinician” was chosen to include healthcare professionals directly involved in patient care, primarily physicians and nurses. This modification ensures that the scale appropriately captures the broader spectrum of interactions patients may have within the healthcare setting for their symptom management, beyond those solely with physicians. Each item was rated on an 11-point Likert scale ranging from 0 (“not at all confident”) to 10 (“very confident”), with a total score range of 0-100. A higher score represented a higher level of perceived communication efficacy in interaction with clinicians. Prior to data collection, an expert panel, comprised of one medical communication specialist, one cancer nurse, and one researcher with expertise in cancer care, reviewed each item for clarity, relevance, and representativeness in relation to the intended construct (Content Validity Index: 1 ). A pilot study involving 100 cancer patients was conducted and confirmatory factor analysis (CFA) indicated a good fit to the data (χ^2^ = 34.78, df = 21, *p* = 0.03 RMSEA = 0.08 GFI = 0.94, IFI = 0.99, TLI = 0.98, CFI = 0.99).

### Statistical analysis

Statistical analysis was performed using SPSS 22.0 (IBM Corp., Armonk, NY). Non-parametric analysis was used to examine the association between participant demographic and clinical characteristics and unmet care needs for symptom management for skewed distribution of unmet needs for symptom management. Spearman correlative analysis was used to explore the correlation between symptom severity, unmet care needs for symptom management, and perceived communication efficacy for the skewed distribution of all these variables. Linear regression analysis was performed, with unmet care needs for symptom management being the dependent variable and perceived communication efficacy being a predictor while controlling for symptom severity and some demographic and clinical variables. All tests were two-tailed, and the criterion for statistical significance was set at *p* < 0.05.

## Results

### Demographic and clinical characteristics

A total of 295 patients with lung and colorectal cancer were approached, of which 73 patients did not complete the survey due to various reasons, resulting in a response rate of 75.3%. Questionnaires with > 10% missing data were excluded, and data from 203 patients were analyzed in this study. Participants were aged between 23 and 80 years (mean = 57.03, SD = 9.71), and 65% were male (Table 1). 52% of participants were diagnosed with lung cancer, and 48% were diagnosed with colorectal cancer (Table 1). A substantial proportion of participants had cancer metastasis (78%) and were receiving more than three cycles of chemotherapy (71%) (Table 1).

**Table 1:** Descriptive statistics of study variables and their association with unmet care needs for symptom management (*N* = 203)

Study variables	n (%)	Unmet care needs for symptom management M (SD)	Z/χ^2^	*p*
Age				
< 60 years old	114 (56.2)	1.46 (3.02)	-1.83	0.068
≥ 60 years old	89 (43.8)	1.55 (2.48)		
Sex				
Male	132(65.0)	1.73 (2.91)	-2.26	0.024
Female	71(35.0)	1.07 (2.52)		
Marital status				
Married	197(97.0)	1.50 (2.80)	-0.44	0.660
Unmarried	6(3.0)			
Level of education		1.33 (2.80)		
Lower than high school	119(58.6)	1.23 (2.30)	-0.81	0.418
High school or higher	84(41.4)	1.88 (3.34)		
Monthly household income				
≤2000 RMB	41(20.2)	2.78 (3.84)	-3.08	0.002
>2000 RMB	162(79.8)	1.17 (2.36)		
Cancer diagnosis				
Lung cancer	106(52.2)	1.69 (2.93)	-1.54	0.124
Colorectal cancer	97(47.8)	1.29 (2.63)		
Cancer stage				
Stage I or II	18 (8.8)	1.61 (3.27)	0.07	0.966
Stage III or IV	137 (67.2)	1.36 (2.59)		
Unclear	48 (24.0)	1.85 (3.14)		
Comorbidities				
0	67(33.0)	1.14 (2.39)	-1.34	0.182
≥1	136(67.0)	1.67 (2.96)		
Cancer metastasized				
No	45(22.2)	1.38 (2.34)	-0.17	0.868
Yes	158(77.8)	1.53 (2.91)		
Duration of chemotherapy				
≤3 cycles	58(28.6)	1.41 (2.88)	− 0.21	0.835
> 3 cycles	145(71.4)	1.53 (2.76)		
Length of hospital stay				
3 days or less	152(74.9)	1.39 (2.52)	− 0.18	0.883
More than 3 days	51(25.1)	1.82 (3.47)		
Symptoms with unmet needs				
0	116 (57.1)	-	-	-
≥ 1	87 (42.9)	-	-	-

### Unmet care needs for symptom management

An average of 5.9 (range: 0–13; SD = 3.69) chemotherapy-induced symptoms were reported by participants during hospitalization, with a symptom severity score ranging between 0 and 90 (mean = 16.36; SD = 17.46) (Table 3). Participants reported an average of 3.2 (range: 0–13; SD = 3.54) symptoms requiring supportive care and 1.5 (range: 0–13; SD = 2.79) symptoms for which their care needs were unfulfilled during their hospital stay (Table 3). Approximately 43% of participants had at least one symptom with unmet needs. Fatigue was reported as the symptom with the highest occurrence (66%), the highest demand for supportive care (36%), and the highest prevalence of unmet needs (19%) (Table 2). Ranked second for symptom occurrence (59%), and the prevalence of unmet needs was decreased appetite (16%) (Table 2). Unmet care needs for symptom management were significantly higher in male participants (*Z*=-2.26; *p* = 0.024) and participants with monthly household income lower than 2000 RMB (*Z*=-3.08; *p* = 0.002) (Table 1). There was no statistically significant difference in unmet care needs between patients with and without comorbidities (Table 1).

**Table 2:** The prevalence of symptom occurrence, and the respective care needs and unmet needs (*N* = 203)

		Symptom occurrence		Symptoms requiring supportive care		Symptoms with unmet needs
Rank		n (%)		n (%)		n (%)
1	Fatigue	134(66.0)	Fatigue	72(35.5)	Fatigue	38(18.7)
2	Decreased appetite	120(59.1)	Pain	61(30.0)	Decreased appetite	32(15.8)
3	Dry mouth	108(53.2)	Nausea	59(29.1)	Numbness	28(13.8)
4	Numbness	102(50.2)	Decreased appetite	58(28.6)	Pain	24(11.8)
5	Sleep disturbance	97(47.8)	Dry mouth	58(28.6)	Dry mouth	24(11.8)
6	Pain	95(46.8)	Numbness	53(26.1)	Nausea	23(11.3)
7	Nausea	95(46.8)	Shortness of breath	52(25.6)	Vomiting	22(10.8)
8	Distress	88(43.3)	Sleep disturbance	51(25.1)	Shortness of breath	21(10.3)
9	Shortness of breath	83(40.9)	Vomiting	51(25.1)	Sleep disturbance	20(9.9)
10	Vomiting	72(35.5)	Distress	48(23.6)	Drowsiness	20(9.9)
11	Drowsiness	72(35.5)	Drowsiness	36(17.7)	Distress	19(9.4)
12	Sadness	64(31.5)	Sadness	28(13.8)	Memory problems	17(8.4)
13	Memory problems	63(29.6)	Memory problems	28(13.8)	Sadness	16(8.4)

### The association between perceived communication efficacy and unmet care needs

The average score of communication efficacy perceived by patients with cancer in this study was 74.04 (SD = 19.12, ranging from 16 to 100) (Table 3). Spearman correlation analyses indicated that unmet care needs were significantly negatively correlated with perceived communication efficacy (*r*=-0.17, *p* < 0.05) and significantly positively correlated with symptom severity (*r* = 0.58, *p* < 0.001) (Table 3). When the association between perceived communication efficacy and unmet care needs was further explored by linear regression analysis, it was evident that low levels of perceived communication efficacy significantly predicted unmet care needs, either in the unadjusted model (*β*=-0.26, *p* < 0.001) or after controlling for household income and symptom severity (*β*=-0.13, *p* = 0.011) (Table 4).

**Table 3:** Spearman correlation matrix between perceived communication efficacy, symptom severity, and unmet needs for symptom management (*N* = 203)

	M (SD)	Range	Perceived communication efficacy	Symptom severity	Unmet needs for symptom management
Perceived communication efficacy	74.04 (19.12)	16–100	1		
Symptom severity	16.36 (17.46)	0–90	-0.18*	1	
Unmet needs for symptom management	1.50 (2.79)	0–13	-0.17*	0.58**	1

Note. *p < 0.05,**p < 0.001

**Table 4:** Linear regression analyses of the association between perceived communication efficacy and unmet needs for symptom management (*N* = 203)

	Model 1						Model 2						
Variables	B	95% CI	SE	β	t	*p*		B	95% CI	SE	β	t	*p*
Perceived communication efficacy	-0.04	-0.06, -0.02	0.01	-0.26	-3.81	< 0.001		-0.02	-0.03, -0.00	0.01	-0.13	-2.57	0.011
Symptom severity								0.10	0.09, 0.12	0.01	0.64	12.68	< 0.001
Sex								-0.50	-1.07, 0.07	0.29	-0.09	-1.72	0.087
Monthly household income								-0.66	-1.35, 0.03	0.35	-0.10	-1.90	0.059

Note. CI = confidence intervals. In Model 1, R^2^ = 0.07, F = 14.50, *p* < 0.001; In Model 2, symptom severity and monthly household income were controlled, R^2^ = 0.52, F = 53.32, *p* < 0.001. ΔR^2^ = 0.45 between Model 1 and Model 2

## Discussion

This study examined the unmet care needs for the management of chemotherapy-related symptoms in patients with lung and colorectal cancers and their association with patient-perceived communication efficacy. Approximately 43% of the study sample reported at least one symptom for which the supportive care needs were unfulfilled, fatigue and decreased appetite being the most prevalent. Perceived low levels of communication efficacy independently predicted unmet care needs for symptom management.

The prevalence of unmet care needs for symptom management found in this study is markedly higher than that reported by Walling et al. (15%).^14^ Walling et al.’s study population was newly diagnosed lung and colorectal patients admitted to a primary care setting, while our study was conducted on relatively advanced-stage lung and colorectal patients admitted to an acute-care setting. Symptom occurrence and severity are proportional to cancer stage and can affect supportive care needs, [36] which may partly explain the observed prevalence discrepancy in the two studies. Additionally, the varied assessment tools utilized in these two surveys may also account for the difference. In the present study, although the majority of patients had advanced diseases, the average number of symptoms for which their care needs were unfulfilled was relatively low (1.5). The small mean unmet needs may be explained by several factors. Firstly, when participants were hospitalized, clinicians were actively involved in providing care to address a significant portion of their symptom-related needs. Secondly, protocols for symptom management in acute-care settings are often characterized by a more comprehensive approach, addressing a broader spectrum of symptoms [37].

Consistent with earlier research findings, [14, 18, 19, 38] fatigue and decreased appetite were identified as the most commonly reported symptoms with unmet needs among patients with colorectal and lung cancer. These two symptoms are widely known as common side effects of chemotherapy and can last throughout the cancer treatment journey and even after treatment completion [18, 19]. Previous studies have also shown that fatigue is often underreported by patients and undertreated by physicians [39]. Barriers to effective management of fatigue included poor awareness of the impact of fatigue on quality of life and lack of clinical guidelines for fatigue management [40, 41]. According to the 2023 National Comprehensive Cancer Network (NCCN) Clinical Practice Guidelines in Oncology, physical exercise, yoga, and mindfulness-based interventions are recommended for patients with cancer experiencing fatigue [42]. Despite the importance of introducing these interventions clinically, there is a lack of clinical guidelines as well as data to inform such practice, particularly in the acute care setting [43]. Similarly, decreased appetite has also been reported as a debilitating chemotherapy-triggered symptom but is often underappreciated and undermanaged in cancer care [44]. Although poor appetite inducing malnutrition has been widely recognized, current cancer supportive care regimens specifically targeting chemotherapy-related appetite loss are sparse and unavailable to many remote and regional settings [44–46]. 

This study also found that perceived communication efficacy by patients with cancer was an independent predictor of unmet care needs. This finding mirrored those of previous studies [14, 32]. Poor patient‒physician communication leading to higher symptom severity and more unfulfilled supportive care needs has been demonstrated in patients with breast cancer and lung and colorectal cancer [14, 32]. As symptoms are subjective experiences and rely on the effective and accurate conveyance of personal feelings, it requires both patients and clinicians to be mutually engaged in the conversation and to be in a trusting relationship for sharing private information. This is particularly essential in cancer care given that the psychological distress caused by unsatisfied care demands may exacerbate perceived symptom severity [47]. Indeed, in our study, the self-reported severity of symptoms in patients with cancer was significantly associated with unmet care needs for symptom management, as well as perceived communication efficacy. These findings indicate the need for the development of clinical programs that actively engage and empower patients with cancer to voice their feelings and needs to healthcare professionals. A patient-centered communication approach that fosters respect and encourages information sharing should be adopted. Additionally, clinical training programs that strengthen core communication skills in clinicians such as nurses and physicians and other healthcare professionals in direct patient-facing roles are also crucial.

Our study showed that male patients had more unmet care needs for symptom management than female patients, which is inconsistent with previous study conducted among patients with head and neck cancer [48]. The inconsistent results between these two studies can be explained by different cancer type. In line with prior research findings, [49, 50] our study found that unmet care needs for symptom management were significantly inversely associated with household incomes in patients with cancer. This finding is unsurprising, as low-socioeconomic-status patients with cancer tend to choose cheaper chemotherapy regimens and thus may suffer from a heavier symptom burden [51, 52]. Coverage of cancer treatment expenditure, particularly for low-income community members, is not instituted in China [53]. However, such a need is indicated, as it will significantly improve the quality of life of patients with cancer by relieving chemotherapy-related symptoms that otherwise have to be endured.

### Limitations

Several limitations need to be noted. Firstly, participants were exclusively recruited from a single hospital in China by convenience sampling, with a significant majority being advanced-stage patients (stage III and IV). This may affect the generalizability of findings to the broader population of lung and colorectal cancer patients. Sampling across a greater geographic area and involving multiple clinical settings are needed to produce more robust and representative data. Secondly, the study’s focus on examining symptom burden of lung cancer and colorectal cancer chemotherapy collectively may introduce potential data bias due to dissimilarities in chemotherapy regimens and associated side effects for the two cancer types. Thirdly, the survey tool used for communication efficacy assessment, while validated for the present study sample, is not specific to the cancer care context. The modification from ‘physician’ to ‘clinician’ in the survey tool may obscure potential nuances in patient communication with physicians versus nurses, given their distinct roles in symptom management. Our findings are thus more likely to reflect the overall communication dynamics between patients and this broader group of healthcare professionals. Fourthly, the cross-sectional design is limited in determining a causal relationship between perceived communication efficacy and unmet care needs. Future longitudinal studies may be undertaken to further explore causality between these two study variables.

## Conclusions

Unmet needs for chemotherapy-associated symptom management are prevalent in patients with lung and colorectal cancer in China, with lower socioeconomic status patients disproportionately affected. Perceived communication efficacy is an independent predictor of unmet care needs. This study highlights the necessity of introducing clinical assessment tools and guidelines to address fatigue and other chemotherapy-induced symptoms in patients with lung and colorectal cancer. A patient-centered communication approach considering patients’ culture needs such as collectivist culture and social stigma is recommended for clinicians to engage patients with cancer to voice their needs. Clinical programs designed to strengthen patients’ communication efficacy especially for patients with special culture or social are also warranted.

## Data Availability

The data and materials are available from corresponding author on reasonable request.
